# Measuring semantic similarities by combining gene ontology annotations and gene co-function networks

**DOI:** 10.1186/s12859-015-0474-7

**Published:** 2015-02-14

**Authors:** Jiajie Peng, Sahra Uygun, Taehyong Kim, Yadong Wang, Seung Y Rhee, Jin Chen

**Affiliations:** 1 School of Computer Science and Technology, Harbin Institute of Technology, Harbin, China; 2 Department of Energy Plant Research Laboratory, Michigan State University, East Lansing, MI 48824 USA; 3 Genetics Program, Michigan State University, East Lansing, MI 48824 USA; 4 Department of Plant Biology, Carnegie Institution for Science, 260 Panama St, Stanford, CA 94305 USA; 5 Department of Computer Science and Engineering, Michigan State University, East Lansing, MI 48824 USA

**Keywords:** Co-Function network, Gene ontology, Semantic similarity, Gene function annotation

## Abstract

**Background:**

Gene Ontology (GO) has been used widely to study functional relationships between genes. The current semantic similarity measures rely only on GO annotations and GO structure. This limits the power of GO-based similarity because of the limited proportion of genes that are annotated to GO in most organisms.

**Results:**

We introduce a novel approach called *NETSIM* (network-based similarity measure) that incorporates information from gene co-function networks in addition to using the GO structure and annotations. Using metabolic reaction maps of yeast, Arabidopsis, and human, we demonstrate that *NETSIM* can improve the accuracy of GO term similarities. We also demonstrate that *NETSIM* works well even for genomes with sparser gene annotation data. We applied *NETSIM* on large Arabidopsis gene families such as cytochrome P450 monooxygenases to group the members functionally and show that this grouping could facilitate functional characterization of genes in these families.

**Conclusions:**

Using *NETSIM* as an example, we demonstrated that the performance of a semantic similarity measure could be significantly improved after incorporating genome-specific information. *NETSIM* incorporates both GO annotations and gene co-function network data as a priori knowledge in the model. Therefore, functional similarities of GO terms that are not explicitly encoded in GO but are relevant in a taxon-specific manner become measurable when GO annotations are limited. Supplementary information and software are available at http://www.msu.edu/~jinchen/NETSIM.

**Electronic supplementary material:**

The online version of this article (doi:10.1186/s12859-015-0474-7) contains supplementary material, which is available to authorized users.

## Background

**Figure 1:**
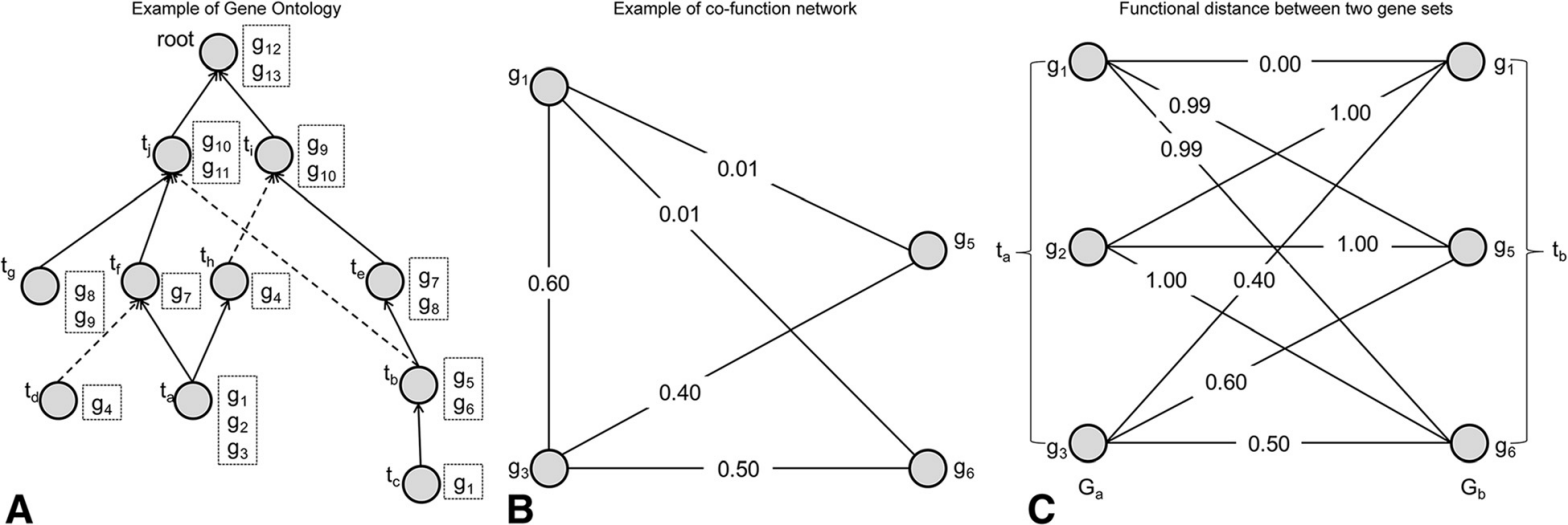
**An example of GO structure and annotation, gene co-function network, and the functional distance. (A)** GO structure and annotation. t_a_…t_j_ and “*root*” are GO terms, edges are the *‘is-a’* (solid line) or *‘part-of’* (dashed line) relations between these terms, and *{g*_*1*_ *…g*_*13*_ *}* in boxes are the sets of genes annotated to the corresponding terms. **(B)** An example of a co-function network. Each node and edge represents a gene and a functional association between the genes, respectively. The number at each edge represents a confidence score that measures the probability of an interaction to represent a true functional linkage between the genes. **(C)** An example of the functional distance between two gene sets. *G*_*a*_ (or *G*_*b*_) is the set of genes annotated to *t*_*a*_ (or *t*_*b*_) or its descendants. The number at each edge represents the functional distance between the genes where 0 = functional identity and 1 = no functional relationship.

Gene Ontology (GO) is a popular vocabulary system for systematically describing the attributes of biological entities in three key domains that are shared by all organisms: molecular function (e.g. biochemical function of the gene product), biological process (e.g. the biological goal to which the gene product contributes) and cellular component (e.g. location of the gene product in the cell) [1]. In each domain, the ontology is structured as a directed acyclic graph to reflect the complex hierarchy of biological events and locations (Figure 1A). Functional analysis based on the similarity of GO terms can lead to new insights about gene functional studies [2], such as gene clustering [3], high-throughput data quality assessment [4], and gene function inference [5,6].

Despite considerable progress on GO based semantic measurements [7-13], our understanding of the semantic relationships between GO terms is still limited. For example, the process ‘response to water deprivation’ (GO:0009414) is intimately related to the following processes in plants: photosynthesis (GO:0015979); anthocyanin biosynthesis (GO:0009718); stomatal closure (GO:0090332); leaf development (GO:0048366); and root development (GO:0048364). However, none of these terms are linked to ‘response to water deprivation’ in GO nor should they be, since these processes are not likely to be associated with ‘response to water deprivation’ in non-plant organisms.

By combining GO with genome specific information such as the co-occurrences of GO annotations in genomes [14], similarities between functions with genome-specific relationships, which are not explicitly represented in GO, may be more accurately scored. We hypothesize that by incorporating a co-function network of genes as additional biological knowledge, we can develop novel insights into GO term relationships. A gene co-function network is built with omics data using a confidence score that measures the probability of an interaction to represent a true functional linkage between two genes based on benchmarked data (Figure 1B) [15-17].

To test this hypothesis, we developed a novel approach called *NETSIM* (network-based similarity measure) that measures functional similarities between GO terms by incorporating information from gene co-function networks in addition to using the GO structure and annotations. Semantic similarity measures have been applied on GO [7-11]. However, these approaches are limited to the topology of the GO structure and the number of annotations to GO terms [14,18]. To date, most genomes have limited functional annotations. For example, only 37% of the Arabidopsis genes are annotated to all three domains of GO based on experimental evidence [19]. Consequently, these approaches may suffer from the limited knowledge encoded in the GO and the limited amount of annotations to GO for most organisms. *NETSIM* addresses these problems by incorporating gene co-function networks in measuring GO-term based similarities.

Using metabolic reaction maps of yeast, Arabidopsis, and human, we demonstrate that GO term relationships can be better scored by incorporating additional biological information from gene co-function networks. We also demonstrate that *NETSIM* works well even for genomes with sparser gene annotation data. We applied *NETSIM* on large Arabidopsis gene families such as cytochrome P450 monooxygenases to group the members functionally and show that this grouping could facilitate functional characterization of genes in these families.

## Methods

### NETSIM: A new approach to study GO term relationships

*NETSIM* measures the similarity between a pair of GO terms within a category in three steps. First, it calculates the functional distance between a pair of gene sets that are annotated to a pair of GO terms using a gene co-function network. Second, it calculates GO term similarity based on the annotations to the common parent term, but propagates only the annotations to the terms that lie on the paths from the two GO terms to the common parent term. Third, it computes similarity between the two GO terms based on the functional distance of annotated genes from co-function networks and the path-constrained GO annotation.

#### Step 1: Functional distance between gene sets

Given any two terms *t*_*a*_ and *t*_*b*_ within a GO category (*e.g.* in Figure 1A), we define the “Gene Set Distance” *D(t*_*a*_ *, t*_*b*_ *)* to measure the relationship between the sets of genes annotated to *t*_*a*_ and *t*_*b*_ in a gene co-function >network by a modified *Czekanovski-Dice* distance [20]. The Gene Set Distance *D(t*_*a*_ *, t*_*b*_ *)* represents the functional distance between two GO terms *t*_*a*_ and *t*_*b*_ based on the functional distances of their annotated gene sets *G*_*a*_ and *G*_*b*_ in the gene co-function network. *D(t*_*a*_ *, t*_*b*_ *)* is defined as:

1 $$ \mathrm{D}\left({\mathrm{t}}_{\mathrm{a}},{\mathrm{t}}_{\mathrm{b}}\right)=\frac{{\displaystyle {\sum}_{g_i\in {G}_a}}{\displaystyle {\prod}_{g_j\in {G}_b}}{d}_{ij}+{\displaystyle {\sum}_{g_i\in {G}_b}}{\displaystyle {\prod}_{g_j\in {G}_a}}{d}_{ij}}{2\left|{G}_a{\displaystyle \cup }{G}_b\right|-{\displaystyle {\sum}_{g_i\in {G}_a}}{\displaystyle {\prod}_{g_j\in {G}_b}}{d}_{ij}-{\displaystyle {\sum}_{g_i\in {G}_b}}{\displaystyle {\prod}_{g_j\in {G}_a}}{d}_{ij}} $$

where *G*_*a*_ ∪ *G*_*b*_ is the union of *G*_*a*_ and *G*_*b*_, ${\displaystyle {\prod}_{g_j\in {G}_b}}{d}_{ij}$ represents the distance between *g*_*i*_ and gene set *G*_*b*_. For any two genes *g*_*i*_ and *g*_*j*_ in a co-function network *NET, d*_*ij*_ is defined as:

2 $$ {d}_{ij}=\left\{\begin{array}{l}0;\kern9.25em i=j\\ {}1;\kern1em <{g}_i,{g}_j>\notin NET\  and\ i\ne j\\ {}1- conf\left({g}_i,{g}_j\right);\kern3.5em  else\end{array}\right. $$

where *< g*_*i*_ *, g*_*j*_ *>* is an edge between *g*_*i*_ and *g*_*j*_ in a gene co-function network *NET*, and *conf(g*_*i*_ *, g*_*j*_ *)* is the confidence score of edge *< g*_*i*_ *, g*_*j*_ *>* in *NET*, *conf*(*g*_*i*_, *g*_*j*_) ∈ [0, 1]. In the illustrative example in Figure 1C, the distance between *g*_*1*_ and *G*_*b*_ is ${\Pi}_{g_j\in {G}_b}{d}_{1\mathrm{j}}=0\times 0.99\times 0.99=0$, indicating *g*_*1*_ and *G*_*b*_ are strongly associated because *g*_*1*_ is one of the genes in *G*_*b*_. Likewise, because *g*_*2*_ does not connect to any of the genes in *G*_*b*_ in the gene co-function network, the distance between *g*_*2*_ and *G*_*b*_ is ${\Pi}_{g_j\in {G}_b}{d}_{2\mathrm{j}}=1\times 1\times 1=1$, meaning that *g*_*2*_ is not related to *G*_*b*_.

#### Step 2: Path-constrained annotation

In the LCA (lowest common ancestor)-based measures [7,8], all of the descendants of LCA are considered. However, any term that lies outside of the paths leading from the terms being compared to the LCA may not directly contribute to the similarity of the two terms. To relate terms to each other more specifically, we defined “Path-Constrained Annotation” *U(t*_*a*_ *,t*_*b*_ *,p)* to use a subset of the terms most relevant to the terms being compared, *i.e.,* the union of three subsets: the genes annotated to the two given terms *t*_*a*_ and *t*_*b*_, and the genes annotated to a common parent term *p* and its descendants that reside only on the paths from *t*_*a*_ or *t*_*b*_ to the parent term.

In the example given in Figure 1A, *t*_*i*_ is the LCA of *t*_*a*_ and *t*_*b*_. To measure the similarity between *t*_*a*_ and *t*_*b*_, *NETSIM* considers only the most relevant terms rather than counting all of the descendants of the LCA, *i.e., {t*_*a*_ *, t*_*b*_ *, t*_*c*_ *, t*_*e*_ *, t*_*h*_ *, t*_*i*_ *}*, as all other LCA based measures do. For the common ancestor *t*_*i*_, *NETSIM* counts the terms on the path from *t*_*a*_ or *t*_*b*_ to *t*_*i*_ and all the descendants of *t*_*a*_ and *t*_*b*_, *i.e., {t*_*a*_ *, t*_*b*_ *, t*_*c*_ *, t*_*e*_ *, t*_*h*_ *, t*_*i*_ *}*. Similarly, for the common ancestor *t*_*j*_, *NETSIM* counts *{t*_*a*_ *, t*_*b*_ *, t*_*c*_ *, t*_*f*_ *, t*_*j*_ *}*_*.*_Therefore, *U(t*_*a*_ *, t*_*b*_ *, t*_*j*_ *) = {g*_*1*_ *, g*_*2*_ *, g*_*3*_ *, g*_*5*_ *, g*_*6*_ *, g*_*7*_ *, g*_*10*_ *, g*_*11*_ *}* does not contain any genes annotated to *t*_*d*_ or *t*_*g*_. Removing such genes reduces less relevant information and helps increase the performance of a term similarity measure.

#### Step 3: Term-to-Term similarity

Given two GO terms *t*_*a*_ and *t*_*b*_ and their common ancestor *p*, the *NETSIM* similarity between the two terms, *S(t*_*a*_ *, t*_*b*_ *, p),* is defined as:

3 $$ \mathrm{S}\left({\mathrm{t}}_{\mathrm{a}},{\mathrm{t}}_{\mathrm{b}},\mathrm{p}\right)=\frac{2 \log \left|G\right|-2 \log f\left({t}_a,{t}_b,p\right)}{2 \log \left|G\right|-\left( \log \left|{G}_a\right|+ \log \left|{G}_b\right|\right)}\times \left(1-\frac{h\left({t}_a,{t}_b\right)}{\left|G\right|}\times \frac{\left|{G}_p\right|}{\left|G\right|}\right) $$

where *G*_*p*_ (or *G*) is the set of genes annotated to *p* (or the root term) and its descendants, *f(t*_*a*_ *, t*_*b*_ *, p)* measures the importance of the path-constrained annotation, and *h(t*_*a*_ *, t*_*b*_ *)* weights the specificity of the common parent *p*:

4 $$ \mathrm{f}\left({\mathrm{t}}_{\mathrm{a}},{\mathrm{t}}_{\mathrm{b}},\mathrm{p}\right)=\mathrm{D}{\left({\mathrm{t}}_{\mathrm{a}},{\mathrm{t}}_{\mathrm{b}}\right)}^2\times \left|U\left({t}_a,{t}_b,p\right)\right|+\left(1-\mathrm{D}{\left({\mathrm{t}}_{\mathrm{a}},{\mathrm{t}}_{\mathrm{b}}\right)}^2\right)\times \sqrt{\left|{G}_a\right|\times \left|{G}_b\right|} $$

and

5 $$ \mathrm{h}\left({\mathrm{t}}_{\mathrm{a}},{\mathrm{t}}_{\mathrm{b}}\right)=\mathrm{D}{\left({\mathrm{t}}_{\mathrm{a}},{\mathrm{t}}_{\mathrm{b}}\right)}^2\times \left|G\right|+\left(1-\mathrm{D}{\left({\mathrm{t}}_a,{\mathrm{t}}_b\right)}^2\right)\times \max \left(\left|{G}_a\right|,\left|{G}_b\right|\right) $$

In Equation 3, the first part measures the relative distance from *t*_*a*_ and *t*_*b*_ to *p*, and the second part (representing the specificity of *p*) measures the distance from the root to *p* in the GO structure. Unlike the existing measures, *NETSIM* incorporates both GO and gene co-function network data. In Equation 4, *D(t*_*a*_ *, t*_*b*_ *)* measures the functional distance between two sets of genes annotated to *t*_*a*_ and *t*_*b*_ in the gene co-function network. If the two sets of genes are not tightly associated in the gene co-function network, *D(t*_*a*_ *, t*_*b*_ *)* is close to 1, leading to small similarity scores. Equation 5 measures the effect of *D(t*_*a*_ *, t*_*b*_ *)* on the distance between *p* and the root. If *D(t*_*a*_ *, t*_*b*_ *)* is close to 1, then *h(t*_*a*_ *, t*_*b*_ *)* is close to *|G|*, resulting in a shorter distance between *p* and the root. If *D(t*_*a*_ *, t*_*b*_ *)* is close to 0, then *h(t*_*a*_ *, t*_*b*_ *)* is close to *max(|G*_*a*_ *|, |G*_*b*_ *|)*, leading to longer distance between *p* and the root.

Mathematically, the Schlicker and Resnik measures [7,8] are two special cases of *NETSIM*. When the gene co-function network data is not available, *G*_*a*_ ∩ *G*_*b*_ ≠ ∅, and *U(t*_*a*_ *, t*_*b*_ *, p) = G*_*p*_. Therefore, *D(t*_*a*_ *, t*_*b*_ *) = 1* and S(t_a_, t_b_, p) = 2IC(p)/(IC(t_a_) + IC(t_b_)) × (1 − |G_p_|/|G|), which is identical to the Schlicker measure. On the other hand, if *t*_*a*_ *= t*_*b*_, then *G*_*a*_ *= G*_*b*_ and *D(t*_*a*_ *, t*_*b*_ *) = 0*. Therefore, *S*(*t*_*a*_, *t*_*b*_, *p*) = 1 − |*G*_*p*_|^2^/|*G*|^2^, which is proportional to the Resink measure where *S(t*_*a*_ *, t*_*b*_ *, p)* is determined solely by the specificity of *p* in the GO structure.

### NETSIM implementation and data preparation

NETSIM was implemented with Java JDK 1.6 and JUNG library (jung.sourceforge.net) [21] (see Additional file 1 for pseudo code and efficiency improvement). GO data was downloaded from the GO website in June 2011 (http://www.geneontology.org/GO.downloads.shtml). In this paper, we used non-IEA annotations for performance evaluation, and only the is-a and part-of relationships were used. In the software, the user can choose to include IEA annotations.

Gene co-function networks were downloaded from YeastNet (http://www.functionalnet.org/yeastnet) [15], AraNet (http://www.functionalnet.org/aranet) [16], and HumanNet (http://www.functionalnet.org/humannet) [17] in July 2011. YeastNet has 102,803 linkages among 5,483 yeast genes, AraNet has 1,062,222 linkages among 19,647 genes and HumanNet has 476,399 linkages among 16,243 genes.

The metabolic networks were constructed by extracting reactions, enzymes, genes, and compounds from metabolic pathway databases YeastCyc 15.0 (993 reactions) [22], AraCyc 8.0 (2689 reactions) [23], and HumanCyc 16.1 (2140 reactions) [24]. All three databases were generated using the PathoLogic software, which does not rely on GO annotations [25]. Briefly, proteins that are annotated to Enzyme Commission (EC) numbers are used as input to predict pathways from a reference pathway database called MetaCyc [26]. For HumanCyc, the enzyme data were compiled from the Ensembl database, the LocusLink database and GenBank [24]. AraCyc annotations are derived from manual curation of experimental data or based on sequence-similarity to reference enzyme sequences [23]. Although the initial YeastCyc build was based on GO molecular function annotations, each pathway in YeastCyc has been manually reviewed and the predicted pathways that did not have any experimental evidence were removed (http://pathway.yeastgenome.org/about/YeastCyc_overview.shtml).

We converted the reactions in the pathway databases into metabolic networks by connecting the reactions if they shared at least one compound. We did not use 24 currency compounds to reconstruct the metabolic network because they create biologically unrealistic shortcuts on link-based analyses by interconnecting many reactions in the network (Additional file 2). We then created a gene map by pairing genes if their encoding enzymes catalyzed the same reaction or directly adjacent reactions in the metabolic network.

We downloaded high-confidence genetic interaction (GI) network of yeast containing 194 positive GIs and 529 negative GIs (http://www.utoronto.ca/boonelab/data/szappanos) [27]. We built two non-GI sets by randomly choosing 194 and 529 non-GI pairs from the same website which have the same number of gene pairs as the positive GI set and negative GI set respectively, retaining the same portion of the GI pairs belonging to the same pathway in YeastCyc (Additional files 3 and 4). Arabidopsis gene family cytochrome P450 monooxygenases (P450) [28] was downloaded from TAIR (http://www.arabidopsis.org/browse/genefamily/). Receptor-like kinase gene families (RLK) were downloaded from [29], and transcription factor families (TF) were downloaded from Plant TFDB (http://planttfdb.cbi.pku.edu.cn/) [30].

### Performance evaluation criteria

While there are numerous methods to evaluate gene-to-gene relationships [31], there is still no objective way to assess GO term-to-term relationships. Therefore, we evaluated the performance of *NETSIM* using biological knowledge at the gene level. The same evaluation method is also used in the existing GO term similarity assessments such as Schlicker *et al.* and Wang *et al.* [8,9]. Specifically, for all the measures to compare, we adopted the same method to aggregate the term-to-term similarities to gene-to-gene similarities and compared them with the prior knowledge.

Once the similarities between all GO term pairs have been computed in an organism, the functional similarity between any two genes can be calculated based on the aggregation of the similarities between the GO terms that are annotated to them. Given two genes *g*_*i*_ and *g*_*j*_ and their GO term annotation sets *T*_*i*_ and *T*_*j*_, we compute the gene-to-gene similarity using the *leave-one-out* approach to avoid the circular use of data. For example, to compute the gene similarity between *g*_*6*_ and *g*_*7*_ in Figure 1A, we first remove the two genes from the gene sets annotated to terms *t*_*b*_, *t*_*e*_, *t*_*f*_. The gene sets that are annotated to terms *t*_*b*_, *t*_*e*_, *t*_*f*_ are then *{g*_*1*_ *, g*_*5*_ *}*, *{g*_*1*_ *, g*_*5*_ *, g*_*8*_ *}*, and *{g*_*1*_ *, g*_*2*_ *, g*_*3*_ *, g*_*4*_ *}* respectively. To compute the gene similarity, we aggregate all the relevant term similarities by adopting the gene similarity measure in [9]:

6 $$ \mathrm{G}\mathrm{S}\left({\mathrm{g}}_{\mathrm{i}},{\mathrm{g}}_{\mathrm{j}}\right)=\frac{{\displaystyle {\sum}_{t\in {T}_i}}Sim\left(t,{T}_j\right)+{\displaystyle {\sum}_{t\in {T}_j}}Sim\left(t,{T}_i\right)}{\left|{T}_i\right|+\left|{T}_j\right|} $$

where for each t ∈ T_x_, $\mathrm{S}\mathrm{i}\mathrm{m}\left(\mathrm{t},{\mathrm{T}}_{\mathrm{y}}\right)=ma{x}_{t_y\in {T}_y}S\left(t,{t}_y\right)$ representing the highest similarity between *t* and term set *T*_*y*_, and only the non-zero *Sim(t, T*_*y*_ *)* values are counted. *S(t, t*_*y*_ *)* is the maximum value of *S(t, t*_*y*_ *,p)* for all the common ancestor (*p*) of *t* and *t*_*y*_. The efficiency calculation of *S(t, t*_*y*_ *,p)* is described in Additional file 1.

We evaluated the performance of *NETSIM* by comparing the GO-based distances between the genes in non-adjacent metabolic reactions (inter-distance) and the GO-based distances between the genes in adjacent reactions (intra-distance) in a metabolic reaction map. A biological process such as the synthesis of an amino acid (amino acid biosynthesis pathway) usually contains multiple reactions. The genes in two adjacent reactions should have similar biological process annotations, because the product of one reaction is the substrate of the other reaction, whereas the genes in non-adjacent reactions are more likely to be involved in different biological processes. With this criterion, we evaluated the performance of *NETSIM* by comparing the GO-based distances between the genes in non-adjacent reactions (inter-distance) and the GO-based distances between the genes in adjacent reactions (intra-distance). The difference in the distance between the former and the latter was used as a benchmark to evaluate the performance of the six selected gene similarity measures quantitatively. Mathematically, we borrowed the concept of the logged fold change (LFC) from gene expression studies [32] and defined it as follows: let *g* be a gene involved in the reaction *r*, *G(r)* be set of genes involved in *r*, the LFC score of *r* is computed with following equation:

7 $$ LFC(r)=\frac{{\displaystyle {\sum}_{g\in G(r)}}lfc\left(g,r\right)}{\left|G(r)\right|} $$

where for every gene *g*∈*G(r)*, *lfc(g,r)* is computed as the logged ratio between its distances to every gene in the set of genes involved in adjacent reactions of *r* (*G*_*adj*_ *(r)*) and its distances to every gene in the set of genes involved in non-adjacent reactions of *r*(*G*_*non*_ *(r)*):

8 $$ lfc\left(g,r\right)= ln\frac{1/\left|{G}_{non}(r)\right|\times {\displaystyle {\sum}_{g\hbox{'}\in {G}_{non}(r)}}\left(1-GS\left(g,{g}^{\hbox{'}}\right)+c\right)}{1/\left|{G}_{adj}(r)\right|\times {\displaystyle {\sum}_{g*\in {G}_{adj}(r)}}\left(1-GS\left(g,{g}^{*}\right)+c\right)} $$

where *inter* is the average distance from *g* to every gene in *G*_*non*_ *(r)*, and *intra* is the average distance from *g* to every other gene in *G*_*adj*_ *(r)*, and *c* is a small positive constant. If the LFC score is greater than 0, then the intra-distances are, on average, smaller than the inter-distances. To reduce ambiguity, the genes that belong to both *G*_*adj*_ *(r)* and *G*_*non*_ *(r)* were removed from both *G*_*adj*_ *(r)* and *G*_*non*_ *(r)* before the computation of LFC. Similarly, the overlapping genes between *G(r)* and *G*_*adj*_ *(r)* or between *G(r)* and *G*_*non*_ *(r)* were removed from *G*_*adj*_ *(r)* or *G*_*non*_ *(r)*, respectively. Based on the definition of LFC in Equation 7, the higher the LFC score, the better the corresponding GO gene-to-gene measure is.

### Genome-specificity measurement

In order to test whether adding co-function networks make the GO term similarities more genome-specific, we developed a genome-specificity measurement. First, we generated *NETSIM* term-to-term similarity scores with and without the co-function network. Second, we computed the ratio between the *NETSIM* scores with or without co-function data. Mathematically, given *k* organisms O = *{o*_*1*_ *, o*_*2*_ *, …, o*_*k*_ *}*, we defined the average of GO term similarity difference between any two species as the inter-genome GO similarity difference of term pair (*t*_*i*_ *, t*_*j*_) using co-function network: $Dif{f}_{net}\left({t}_i,{t}_j\right)={\displaystyle \sum_{1\le m,n\le k,m\ne n}\left|{S}_{net}\left({t}_i,{t}_j,{o}_m\right)-{S}_{net}\left({t}_i,{t}_j,{o}_n\right)\right|}.$ Similarly, the inter-genome GO similarity difference of term pair (*t*_*i*_ *, t*_*j*_) without co-function network is $Dif{f}_{nonet}\left({t}_i,{t}_j\right)={\displaystyle \sum_{1\le m,n\le k,m\ne n}\left|{S}_{nonet}\left({t}_i,{t}_j,{o}_m\right)-{S}_{nonet}\left({t}_i,{t}_j,{o}_n\right)\right|}$. The genome-specificity measurement is $Diff\left({t}_i,{t}_j\right)={\displaystyle \sum_{1\le m,n\le k,m\ne n}\left|{S}_{net}\left({t}_i,{t}_j,{o}_m\right)-{S}_{net}\left({t}_i,{t}_j,{o}_n\right)\right|}-{\displaystyle \sum_{1\le m,n\le k,m\ne n}\left|{S}_{nonet}\left({t}_i,{t}_j,{o}_m\right)-{S}_{nonet}\left({t}_i,{t}_j,{o}_n\right)\right|}.$

### Other GO-based semantic similarity measures used for comparison

Mathematically, a GO term is a direct child of another term if and only if the former term is a subtype (*is-a* relationship), a component (*part-of* relationship), or a regulator (*regulates* relationship) of the latter one (e.g. in Figure 1A *t*_*a*_ is a direct child of *t*_*f*_ with *is-a* relationship). The explicitly defined and structured representation of biological terms of GO allows the measurement of similarities between two terms and between two genes that are annotated to the terms.

By combining Information Content (IC) with the ontology structure, Resnik defined a taxonomic similarity as the IC of the lowest common ancestor (LCA) [7], which is then widely used as a similarity measure for GO terms. Let *t* be a GO term, the information content of *t* is defined as *IC(t) = -log(|G*_*t*_ *|/|G|)*, where *G*_*t*_ and *G* are sets of genes annotated to *t* and the root term (and all its descendants). Let *t*_*a*_ and *t*_*b*_ be two GO terms in the same category and *G*_*LCA*_ be the set of gene products annotated to LCA of *t*_*a*_ and *t*_*b*_, the similarity between *t*_*a*_ and *t*_*b*_ is defined as the information content of LCA:

9 $$ {\mathrm{Sim}}_{\mathrm{Resnik}}\left({t}_a,{t}_b\right)=IC(LCA)=- log\frac{\left|{G}_{LCA}\right|}{\left|G\right|} $$

The Resnik measure considers the specificity of the LCA but not the distance from the two terms to their LCA. As a result, pairs of terms that share the same LCA but from different levels of the GO hierarchy can yield the same semantic similarities. Such pairs are therefore not distinguishable from the term pairs that are both close to the LCA. To incorporate distances from two given terms to their LCA and the distance from LCA to the root, Schlicker *et al.* normalized the Resnik measure based on the information content of *t*_*a*_ and *t*_*b*_, and adjusted the overall score with a weighting function:

10 $$ {\mathrm{Sim}}_{\mathrm{Schlicker}}\left({t}_a,{t}_b\right)=\frac{2\times IC(LCA)}{IC\left({t}_a\right)+IC\left({t}_b\right)}\times \left(1-\frac{\left|{G}_{LCA}\right|}{\left|G\right|}\right) $$

The first part of Equation 10 measures the relative distance from *t*_*a*_ and *t*_*b*_ to their LCA, and the second part (weighting function) measures the specificity of LCA in an ontology [8]. Functional similarities of yeast genes using the Schlicker measure correlated well with the established protein sequence similarity approaches [8].

In addition to the LCA based measures, Wang *et al.* proposed a measure that considers the topology of the GO graph by taking into account all of the parent terms (instead of just the LCA), but not the gene annotations [9]. Given a term *t*_*a*_ and its parent term *p* in the GO, the semantic contribution of *p* to *t*_*a*_, denoted as *S*_*ta,p*_, is defined as the maximal semantic contribution of the paths from *t*_*a*_ to *p*. Equation 11 defines the GO term similarity in the Wang measure where *P*_*a*_ (or *P*_*b*_) are the sets of all the parents of *t*_*a*_ (or *t*_*b*_). This measure performed significantly better than the Resnik measure on yeast genes [9].

11 $$ {\mathrm{Sim}}_{\mathrm{Wang}}\left({t}_a,{t}_b\right)=\frac{{\displaystyle {\sum}_{p\in {P}_a{\displaystyle \cap }{P}_b}}\left({S}_{t_{a,p}}+{S}_{t_{b,p}}\right)}{{\displaystyle {\sum}_{t\in {P}_a}}{S}_{t_{a,p}}+{\displaystyle {\sum}_{t\in {P}_b}}{S}_{t_{b,p}}} $$

In summary, the existing GO term similarity measures are purely dependent on the GO structure, and cannot integrate GO with other biological information for providing more accurate term-to-term similarity measures.

## Results and discussion

### Performance evaluation using metabolic reaction maps

We evaluated the performance *NETSIM* by comparing the GO-based distances between the genes annotated to non-adjacent reactions (inter-distance) or between the genes annotated to adjacent reactions (intra-distance) of the metabolic networks of yeast (2,718 links between 546 reactions), Arabidopsis (10,105 links between 1,196 reactions), and human (17,469 links between 1,652 reactions) (Additional files 5, 6 and 7). Reactions with only one gene were removed, resulting in 85 yeast, 493 Arabidopsis, and 379 human reactions. We used logged fold change (LFC) between intra- and inter-distance as a benchmark to compare the performance of six gene similarity measures quantitatively.

We subjected 223 yeast, 1,769 Arabidopsis, and 2,049 human genes with at least one non-IEA GO annotation in the biological process category and associated to a reaction in the metabolic reaction maps to *NETSIM*, previously published measures [7-9,33], and the confidence scores directly obtained from the gene co-function networks to generate gene-to-gene functional similarities.

**Figure 2:**
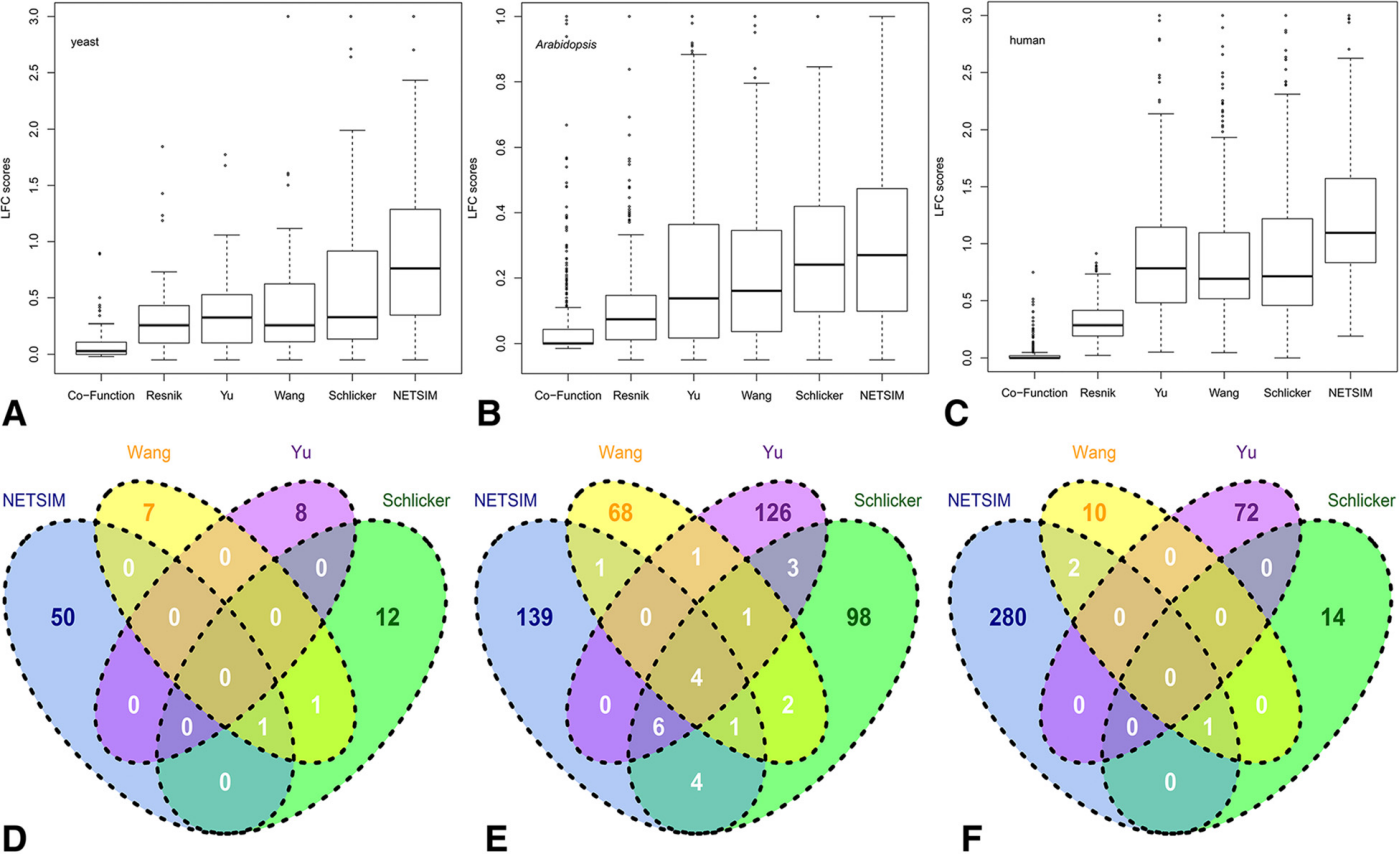
**Performance comparison on Log-transformed Fold Change (LFC) scores of similarity measures on GO’s biological process terms in yeast, Arabidopsis, and human.** Distributions of Log-transformed Fold Change (LFC) scores of similarity measures on GO’s biological process terms in yeast **(A)**, Arabidopsis **(B)**, and human **(C)**. The top and bottom of the boxes represent 75^th^ and 25^th^ percentiles, dark lines are the median, top and bottom whiskers represent greatest and lowest values except outliers. Circles represent outliers that are larger than the sum of 75^th^ and 1.5 interquartile range. Number of reactions for which *NETSIM*, Wang, Yu, and Schlicker measures performed the best for yeast **(D)**, Arabidopsis **(E)**, and human **(F)**.

In all the tests, *NETSIM* performed the best and this improvement was robust to the abundance of gene co-function network data. In yeast, the median LFC score of *NETSIM* was higher than that of all the other measures (Figure 2A, Table 1), indicating that functional similarities determined by *NETSIM* corroborated the best with the reaction map. *NETSIM* also showed the highest first and third quartile LFC scores. Comparing the LFC scores from the six measures for each reaction showed that *NETSIM* performed the best in 50 out of 85 reactions with the Schlicker measure as the runner-up, being the best in 12 reactions (Figure 2D). We also tested *NETSIM* on Arabidopsis and human reaction maps that have fewer GO annotations and smaller and sparser co-function networks. In Arabidopsis, the median LFC score of *NETSIM* was also higher than that of all the other measures (Figure 2B, Table 1). *NETSIM* showed the highest first and third quartile LFC scores, performing the best in 139 out of total 493 reactions, while the Yu measure performs the best in 126 reactions (Figure 2E). For the human data, the median LFC scores of *NETSIM* was 1.10, distinctly higher than the median scores of all other measures (Figure 2C, Table 1). *NETSIM* performed the best in 280 out of 379 reactions while Yu measure was the runner-up, being the best in 72 reactions (Figure 2F). Furthermore, *NETSIM*’s performance was significantly higher from the performances of all the other measures (Tukey multiple comparison test, adjusted p-value < 0.05, Additional files 8 and 9) in all the analyses, except for the Schlicker measure on Arabidopsis. To test the effect of including IEA annotations, we compared all the measures using all the GO annotations including IEA on yeast data. Additional file 10 showed both *NETSIM* and Yu performed better than the other measures on yeast.

**Table 1:** **Comparison of** ***NETSIM*** **’s performance to other similarity measures**

**Similarity**	**Data type used**	**Median (25th Percentile) LFC score**		
**Measure**		**Yeast**	**Arabidopsis**	**human**
co-function	Co-function network	0.03(0.00)	0.00(0.00)	0.00(0.00)
Resnik	GO annotation	0.26(0.10)	0.07(0.01)	0.29(0.19)
Yu	GO annotation	0.32(0.10)	0.14(0.02)	0.78(0.48)
Wang	GO annotation	0.26(0.11)	0.16(0.04)	0.69(0.52)
Schlicker	GO annotation	0.33(0.14)	0.24(0.10)	0.71(0.46)
*NETSIM*	GO annotation,	**0.76(0.35)**	**0.27(0.10)**	**1.10(0.83)**
	co-function network			

Comparison of *NETSIM*’s performance to other similarity measures based on the median and the first quartile of Log-transformed Fold Change (LFC) scores. Numbers in bold indicate the best performance for each species.

In this evaluation, we compared the adjacent and non-adjacent reactions of metabolic networks. However, a biological process, generally composed of larger groups of molecular functions, may contain more than two reactions and therefore genes responsible for close but non-adjacent reactions may also have similar biological process annotations. Therefore, we examined the performance of all the measures between various distances of the reactions. Additional file 11 showed that the averaged LFC scores of all the measures increase proportionally with reaction path length, and *NETSIM* has the highest LFC score at every reaction path length. In summary, *NETSIM* is a reliable term-term measure that performed the best in the majority of the reactions for all three species.

### Performance evaluation using yeast genetic interaction

We further tested the performance of *NETSIM* using genetic interaction (GI) data of yeast metabolic enzymes [27]. A genetic interaction is a phenomenon where mutations in two genes produce a non-additive phenotype in light of each mutation’s individual effects and can reveal functional relationships between genes and pathways [34]. GI can be calculated based on the deviation of the double-mutant phenotype from the product of the corresponding single-mutant phenotypes [27].

**Figure 3:**
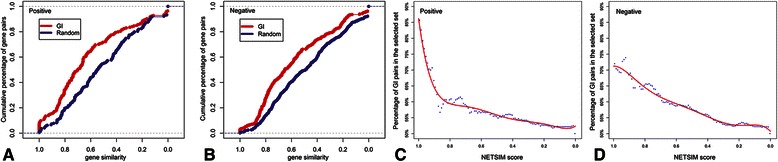
**Performance comparison on yeast genetic interaction data.** Cumulative distributions of *NETSIM*-based gene similarity scores of positive **(A)** and negative **(B)** genetic interactions in yeast. The blue line represents randomly selected ‘non-interaction’ pairs. The red line represents positive or negative genetic interactions. Correlation between *NETSIM*-based gene similarity scores and positive **(C)** or negative **(D)** genetic interactions in yeast. In C and D, the y-axis is percentage of genetic interaction pairs in the selected gene pairs with their *NETSIM* scores larger than a given threshold which varies in the x-axis. The red line is the polynomial model fitting line.

We tested whether the gene-to-gene similarity computed with *NETSIM* would correlate with the GI interaction scores. We used 82 genes that are involved in 32 positive GIs or 28 negative GIs [27], in which two genes in a GI are in the same metabolic pathways in YeastCyc. We also used 307 genes involved in 162 positive GIs or 501 negative GIs, in which two genes in a GI are in different pathways. The distributions of *NETSIM* similarity scores were far from random in both the positive (alleviating) and negative (aggravating) GI sets (p-value 5e-06 (positive GIs vs. random) and p-value 8e-08 (negative GIs vs. random), Kolmogorov-Smirnov test [35], Figure 3A and B). In addition, there is a strong correlation between the genetic interaction and gene-to-gene similarity computed with *NETSIM* for both positive and negative GI pairs (polynomial model with R-squared 0.97 for both positive and negative GI pairs, Figure 3C and D). This suggests that *NETSIM* could be used to predict genetic interactions in genomes that lack genetic interaction information.

### Performance evaluation of each step of *NETSIM*

In order to evaluate the performance of each step of *NETSIM*, we compared *NETSIM* with three versions of *NETSIM*, each with a different approach in step 1, 2 or 3, on yeast data. To test the performance of our co-function integration scheme, we created *NETSIM*_v1where the summed weights of all the edges in a co-function network between two gene sets *G*_*a*_ and *G*_*b*_ were normalized with Equation 12 (to compare with Equation 1). To test the effect of path-constrained annotations, we created *NETSIM*_v2 that uses all the GO annotations without path-based annotation filtering. To test the performance of our scoring scheme in step 3, we created *NETSIM*_v3 that uses a scoring function similar to the Schlicker measure with Equation 13 (to compare with Equation 3):

12 $$ \mathrm{D}\left({\mathrm{t}}_{\mathrm{a}},{\mathrm{t}}_{\mathrm{b}}\right)=\frac{{\displaystyle {\sum}_{g_i\in {G}_a}}{\displaystyle {\sum}_{g_j\in {G}_b}{d}_{ij}}}{\left|{G}_a\right|\times \left|{G}_b\right|} $$

13 $$ \mathrm{S}\left({\mathrm{t}}_{\mathrm{a}},{\mathrm{t}}_{\mathrm{b}},\mathrm{p}\right)=\frac{2 \log \left|G\right|-2 \log f\left({t}_a,{t}_b,p\right)}{2 \log \left|G\right|-\left( \log \left|{G}_a\right|+ \log \left|{G}_b\right|\right)}\times \left(1-\frac{\left|{G}_p\right|}{\left|G\right|}\right) $$

**Figure 4:**
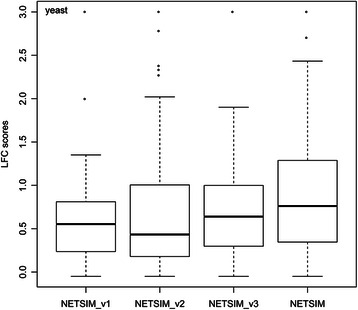
**Performance evaluation by modifying each step of** ***NETSIM*** **.** Three different versions of *NETSIM* (v1, v2 and v3) are compared on yeast. The three versions correspond to the different approaches in NETSIM step 1 Functional Distance between Gene Sets (v1), step 2 Path-Constrained Annotation (v2) and step 3 Term-to-Term Similarity respectively (v3).

Figure 4 shows that *NETSIM* is clearly better than all the three versions, indicating that each step in *NETSIM* contributes to *NETSIM*’s performance and have been appropriately designed. It also shows that the noise reduction by constraining the annotation information (Step 2) is the most important step in improving *NETSIM* performance.

### Effects of *NETSIM* components

*NETSIM* relies on the co-function network, GO structure, and GO annotations. To determine the factors that control *NETSIM*’s performance, we re-ran *NETSIM* by varying the GO annotation and co-function network sizes of yeast, which has the most comprehensive annotation and co-function network. We tested whether changing the gene annotation coverage (number of annotations per GO term) would affect *NETSIM*’s performance by randomly deleting the biological process annotations. To change the gene annotation coverage (number of annotations per GO term), we randomly deleted yeast BP gene annotations. The random process was repeated 1,000 times at each tested coverage level. For every gene we kept at least one annotation during the random deletion process, in order to use the same set of genes to compare different coverage levels. We varied the co-function network density by randomly deleting edges. The random deletion process was repeated 1,000 times at each tested density level. We varied the co-function network quality by randomly swapping the edges in the original network. To generate a ‘low quality’ network, we randomly swapped half of the existing edges in the original network. A fully randomized network was generated by swapping the edges until none of the original edges existed. Each type of network was generated 1,000 times.

**Figure 5:**
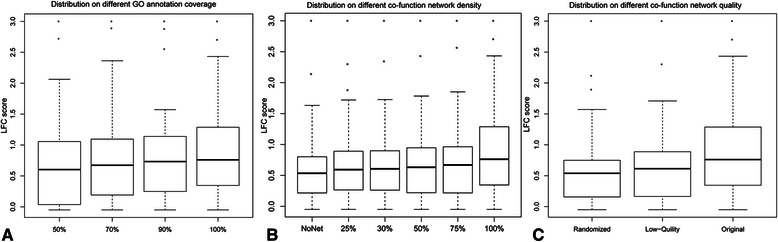
**Distribution of** ***NETSIM*** **-based LFC scores on different input network data.** Distribution of *NETSIM*-based LFC scores using the yeast co-function network and GO biological process annotation data on different GO annotation coverage **(A)**, co-function network density **(B)**, and co-function network quality **(C)**. “Low quality” network means 50% of the links were randomized, and “Randomized” means 100% of the links were randomized.

The performance of *NETSIM* decreased steadily with the reduction of gene annotations, but there was no significant difference among different sizes (p-value > 0.05, Tukey’s multiple comparison test, Figure 5A, Additional file 12). In addition, the median LFC score of *NETSIM* at 50% annotation coverage was still higher than the score from the best available measure (Schlicker), indicating that the co-function network is substitutable to GO annotations to a certain degree. This is important because the gene annotation coverage is usually low for the less-studied genomes, but the co-function networks learned from omics data are rapidly increasing [36].

Since the co-function network was substitutable to GO annotations, we looked for network factors on which the performance of *NETSIM* depended the most. We tested network density (proportion of selected connections to all connections in the network) and network quality (proportion of original connections to randomized connections), by manipulating the yeast co-function network. We varied the yeast co-function network density by randomly deleting the edges from the full net (100%) to no net (0%), and then applied NETSIM on the sparser networks. Decreasing the network density reduced the LFC scores of *NETSIM*. However, the difference in performance was not significant (adjusted p-value > 0.05, Tukey multiple comparison test, Figure 5B and Additional file 12), which indicates that the network density was not a key factor on which the performance of *NETSIM* depended the most. Next, we examined the effect of varying the yeast co-function network quality on *NETSIM*’s performance. The ‘low quality’ network (half of the existing edges swapped) and the fully randomized network affected *NETSIM*’s performance significantly (adjusted p-value < 0.05, Tukey multiple comparison test, Figure 5C and Additional file 12), which indicates that the network quality is a key factor on which the performance of *NETSIM* depended the most. In summary, the co-function network is substitutable to GO annotations to a certain degree and the network quality is a key feature that affects the performance of *NETSIM*. For the less-studied genomes, *NETSIM*, which relies on both the GO infrastructure and co-function network data, can take advantage of both the limited but precious GO knowledge and abundant co-function network data to provide higher quality term-to-term relationships.

To test the extent of information redundancy between GO annotations and co-function networks, we calculated the number of the connected genes in a co-function network that share the same GO annotations, and divided it by the total number of edges in the network. The information redundancy ratio of YeastNet, AraNet, and HumanNet is 51.6%, 35.0% and 43.2%, respectively. This indicates that a co-function network can provide a substantial amount of extra information that is not contained in the GO. This explains why *NETSIM* performance drops significantly if the co-function network is removed from it (Figure 5B). Adding more orthogonal datasets such as physical interaction data [37,38] will likely improve the performance of *NETSIM*.

### Genome-specific GO term relationships

We explored whether using co-function networks not only increase the performance of GO term similarities, but also enables the identification of genome-specific GO term relationships. Genome-specificity of GO term similarity is defined as the difference in GO term similarity between organisms. We generated the *NETSIM* GO similarity scores with and without the co-function network in the three organisms, yeast, Arabidopsis, and human. We then computed the genome-specificity scores for all GO term pairs to test whether adding co-function networks makes the GO term similarities more genome-specific. A GO term pair was deemed genome-specific if its genome-specificity score is significantly different from the averaged genome-specificity score of all GO term pairs. Using t-test, 15,296 significant genome-specific term pairs (FDR < 0.01) were identified (Additional file 13).

### Categorizing Arabidopsis genes using *NETSIM*

Semantic similarity measures have wide-ranging applications, including analyzing clusters of genes and proteins from ‘omics’ experiments [3], assessing the quality of high-throughput data [4], and inferring functions of genes [4]. Here, we used *NETSIM* to functionally categorize Arabidopsis genes belonging to three large families: cytochrome P450 monooxygenases (P450) [28], receptor-like kinase gene families [29], or transcription factor families (TF) [39].

First, *NETSIM* was applied to all gene pairs of Arabidopsis gene family P450 and the resulting similarity matrix was used to generate a similarity tree using hierarchical clustering [40] function called hclust in R (version 2.15) with default parameters. For comparison, a dendrogram for P450 gene family was generated based on protein sequence similarities with the same clustering software. The protein sequence similarities were calculated using bl2seq in BLAST [41].

P450 is a large and diverse group of enzymes involved in many pathways including drug metabolism in animals [42] and specialized metabolism in plants [28]. Despite their importance, only few genes have been characterized experimentally. In Arabidopsis, over 70% of P450s still await functional characterization [28]. We asked whether functional similarities measured with *NETSIM* could help infer functions of the P450s that have not yet been assigned to a specific metabolic pathway.

There are 272 P450 genes in Arabidopsis, 73 of which have non-IEA GO annotations to biological process terms [19]. These 73 genes are grouped into 31 families and 46 subfamilies based on sequence similarity [28]. However, 17 of the 73 genes have not yet been assigned a biochemical function and placed in the Arabidopsis metabolic network AraCyc [43].

**Figure 6:**
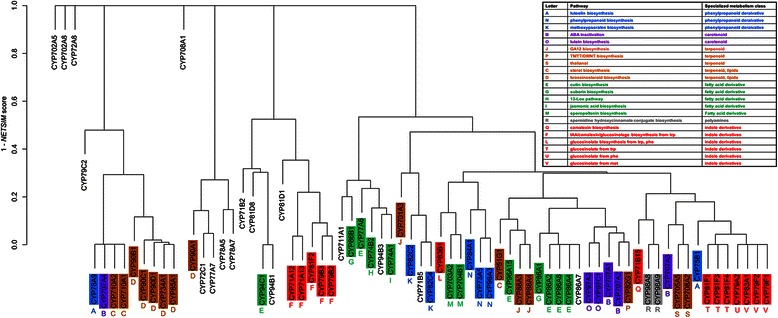
**Hierarchical clustering of Arabidopsis P450 genes with distsances based on** ***NETSIM*** **similarity.** The y-axis is 1 minus *NETSIM*-based gene-to-gene similarity score. The color and letter coding are based on metabolic pathways annotated in AraCyc version 8.0 and are shown in the table.

To infer functions of the 17 genes, we computed functional similarities for all the 73 genes with GO biological process terms using *NETSIM* and grouped them into clusters using a hierarchical clustering algorithm [44] (Figure 6). In addition, a sequence based tree was generated using the same clustering algorithm (Additional file 14). Six types of secondary metabolism are represented in these trees (Figure 6). The function-based and sequence-based trees reveal some similarities but mostly striking differences. Both trees group the genes into four large clusters, but with different members in each cluster. For example, members of the auxin/camalexin/glucosinolate pathways are clustered in the function-based tree (Figure 6, letter F) whereas they are scattered in the sequence-based tree (Additional file 14). Similarly, genes involved in glucosinolate synthesis from tryptophan and methionine are grouped together in the function-based tree whereas they are separated in the sequence-based tree (T and V in Figure 6 and Additional file 14). An exception is brassinosteroid pathway whose members are clustered both in the function-based and sequence-based trees (D in Figure 6 and Additional file 14). In addition, the sequence-based tree typically groups genes belonging to the same biochemical pathway only at the leaf nodes (the two most closely related sequences) whereas the function-based tree shows larger consistencies of grouping members of the same biochemical pathway.

In the function-based P450 tree, 17 genes have not yet been assigned to a metabolic pathway. We can devise different hypotheses from the two trees. For example, CYP81D8 is closest to a known enzyme involved in jasmonic acid metabolism (clustering with CYP94C1 that is involved in this pathway) in the function-based tree, whereas it is most similar to genes involved in glucosinolate metabolism from tryptophan in the sequence-based tree (CYP81F members). Experimental testing of these uncharacterized genes will reveal the true power of these similarity measures to infer function.

**Figure 7:**
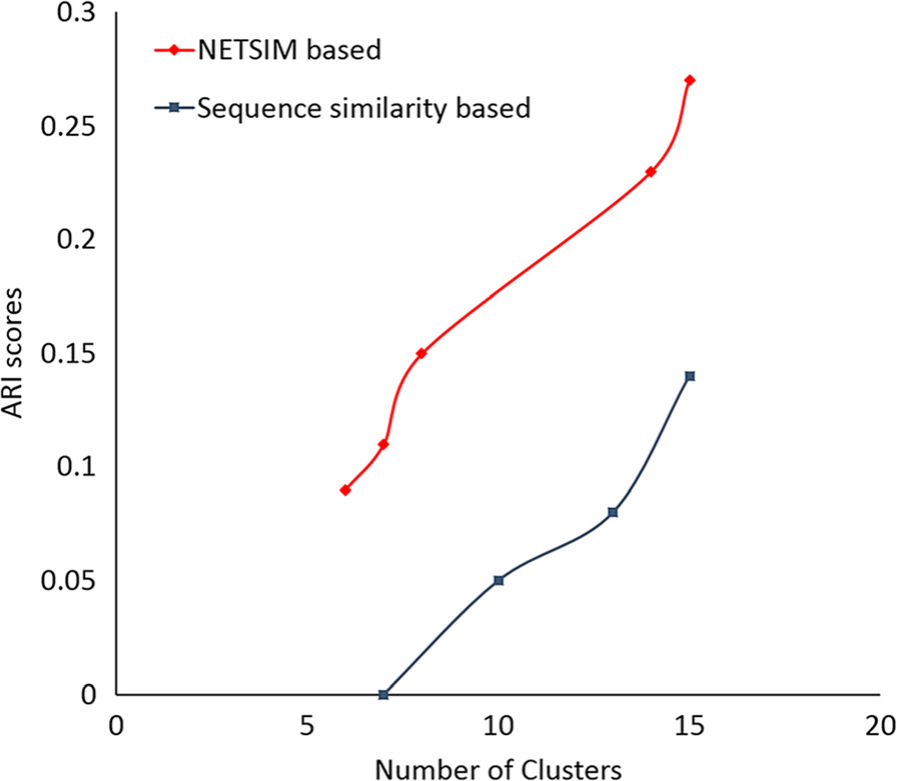
**Adjusted Rand Index (ARI) scores for** ***NETSIM*** **and sequence based similarities.** Comparison of ARI scores for *NETSIM* and sequence based similarities where the x-axis is the number of clusters that have at least two genes, and the y-axis is the ARI scores.

Adjusted Rand Index (ARI) is a frequently used cluster validation measure that measures the amount of agreement between the clusters and some external, often gold-standard, data [45,46]. ARI assumes the generalized hypergeometric distribution as the model of randomness, so that the expected ARI value of two random clustering results is constant (0.0) [45,46]. ARI can therefore be used to compare the results of different clustering methods. Higher ARI values reflect higher cluster quality with respect to the external criteria. In this paper, we used the metabolic pathways that the experimentally characterized P450s belong to as the external criteria to compare *NETSIM* based clustering and sequence similarity based clustering. Figure 7 reveals that the ARI scores of *NETSIM* are consistently higher than the ARI scores of the sequence similarity based clustering results. The best ARI score for *NETSIM* (0.27) is achieved when the number of the *NETSIM* based clusters is 15, clearly higher than the corresponding ARI score for sequence similarity based clusters (0.17). The results indicate that *NETSIM* is better at grouping functions than sequence similarity.

When *NETSIM* was applied to the other two large families RLK (Additional file 15) and TF (Additional file 16), it produced gene clusters that were distinct from sequence-based clusters as well (Additional files 17 and 18). In summary, the application of *NETSIM* on Arabidopsis gene families shows that it can redefine subfamilies by dividing or merging sequence-based subfamilies, which may bring new knowledge in gene function discovery.

## Conclusions

GO annotation data has been used widely to measure functional similarity between genes based on GO term similarities, which helps infer functions of uncharacterized genes. However, existing algorithms only use the GO structure and annotations, both of which have much missing information, leading to less accurate term similarity scores. We developed *NETSIM* based on the notion that incorporating additional biological information may improve the performance of these measures. Incorporation of gene co-function network data clearly helps to improve the performance of the GO term similarity measures when there is abundant gene co-function data.

In comparison with the existing algorithms, *NETSIM* has two advantages. First, both GO annotation and gene co-function network are adopted as *a priori* knowledge in our model, while existing measures incorporate only GO annotations. Therefore, the functional similarities of GO terms that are not explicitly encoded in GO but are relevant in a taxon-specific manner become measurable when GO annotations are limited. Second, only the annotations to the GO terms that lie on the paths from the given terms to the common parent term are propagated, excluding less relevant information from the GO terms that are children of the LCA but branch out from the paths to the LCA. The existing node-based measures propagate all the descendant annotations to the parent.

To demonstrate the advantages of *NETSIM* over the existing measures, we compared *NETSIM* with the Resnik, Schlicker, Wang, and Yu measures. We found that *NETSIM* performed the best in all the tests for yeast, Arabidopsis, and human. We chose these three species for two reasons. First, they have different amounts of annotation; yeast is one of the best annotated organisms based on experimental evidence (85%) [18], while the annotations for Arabidopsis and human (37% and 49%) are still far from saturation [19,47]. Second, they have different sizes and densities of the co-function network data; the density of yeast co-function network is 0.0183 and the network includes 95% of yeast genes [15], while the density of Arabidopsis co-function network is 0.0014 (10-fold lower than that of yeast) and the network includes 73% of Arabidopsis genes [16]. The density of human co-function network is 0.0049 and the network includes 87% of human genes [17].

In summary, using *NETSIM* as an example, we demonstrated that the performance of a semantic similarity measure could be significantly improved after incorporating genome-specific information. *NETSIM* incorporates both GO annotations and gene co-function network data as a priori knowledge in the model. Therefore, functional similarities of GO terms that are not explicitly encoded in GO but are relevant in a taxon-specific manner become measurable when GO annotations are limited.

## Additional files

Additional file 1:
***NETSIM*** **implementation and efficiency improvement.** Describe the pseudocode and efficiency improvement of *NETSIM* including the involved Definition, Lemma, Proof and an example.
Additional file 2:
**24 Currency metabolites that were removed from the metabolic network reconstruction.**

Additional file 3:
***NETSIM*** **similarity scores of positive GI set and a random set.**

Additional file 4:
***NETSIM*** **similarity scores negative GI set and a random set.**

Additional file 5:
**Yeast reaction map.** The yeast reaction map contains 546 reactions and 2,718 links between these reactions.
Additional file 6:
**Arabidopsis reaction map.** The Arabidopsis reaction map contains 1,196 reactions and 10,105 links between these reactions.
Additional file 7:
**Human reaction map.** The human reaction map contains 1,652 reactions and 17,469 links between these reactions.
Additional file 8:
**Tukey test for gene-to-gene similarity on yeast (A), Arabidopsis (B) and human (C).** For each line representing the difference of compared measures, the middle point (triangle) represents the difference in the observed means, and the line itself represents the range of the differences of compared measures.
Additional file 9:
**Adjusted p-value of Tukey multiple comparison test.** Adjusted p-value of Tukey multiple comparison test comparing *NETSIM* with Resnik, Schlicker, Wang and Yu measure on yeast, Arabidopsis and human.
Additional file 10:
**Distributions of Log-transformed Fold Change (LFC) scores of similarity measures.** Distributions of Log-transformed Fold Change (LFC) scores of similarity measures on GO’s biological process (BP) terms in yeast considering all annotations.
Additional file 11:
**The averaged Log-transformed Fold Change (LFC) scores of all the measures at different reaction path lengths on yeast.** The x-axis is the path length to the given reaction. The y-axis is the average of LFC scores for all involved reactions.
Additional file 12:
**The effect of varying GO annotation coverage, co-function network density, and co-function network quality on** ***NETSIM’s*** **performance.** The columns named “Ratio Pair” are two *NETSIM* results with different configurations. The different configurations are different co-function network density, or co-function network quality, or different GO annotation coverages. The numbers in bold type indicate that the two compared *NETSIM* results are significantly different.
Additional file 13:
**Genome specific term pairs.** All the significant genome specific term pairs (FDR adjusted p-value < 0.01) are listed.
Additional file 14:
**Sequence based hierarchical clustering for Arabidopsis P450 gene family.** The color coding is the same as Figure 6. The y-axis is 1 minus sequence-based gene-to-gene similarity score, which is the normalized percent identities value of function bl2seq in BLAST.
Additional file 15:
***NETSIM*** **based clustering for Arabidopsis receptor-like kinase gene families (RLK) gene family.**

Additional file 16:
***NETSIM*** **based clustering for Arabidopsis transcription factor families (TF).**

Additional file 17:
**Sequence based clustering for Arabidopsis receptor-like kinase gene families (RLK) gene family.** The y-axis is 1 minus sequence-based gene-to-gene similarity score, which is the normalized percent identities value of function bl2seq in BLAST.
Additional file 18:
**Sequence based clustering for transcription factor families (TF) gene family.** The y-axis is 1 minus sequence-based gene-to-gene similarity score, which is the normalized percent identities value of function bl2seq in BLAST.
