# Supplementary material for: Measuring semantic similarities by combining gene ontology annotations and gene co-function networks
Source: BMC Bioinformatics. 2015 Feb 14;16:44. doi: 10.1186/s12859-015-0474-7 (PMC4339680; doi:10.1186/s12859-015-0474-7)
Supplement: Additional file 1: — NETSIM implementation and efficiency improvement. Describe the pseudocode and efficiency improvement of NETSIM including the involved Definition, Lemma, Proof and an example. [file 12859_2015_474_MOESM1_ESM.pdf]

Supplementary Document for *Measuring Genome-specific Semantic Similarities Using Gene Ontology and Gene Co-Function Networks*

Section S1. *NETSIM* implementation and efficiency improvement

In order to obtain the maximal score of $S(t_a, t_b, p)$ in Equation 3, the parent term p, which may not necessarily be the lowest common ancestor (LCA), needs to be identified. For example in Figure 1A, t_i is the LCA of t_a and t_b since its information content is the highest among all of the common ancestors. However, the similarity score of t_a and t_b via t_i (0.687) is lower than the score of t_a and t_b via t_j (0.691), which is a non-LCA common ancestor. A simple approach to identify the parent term p that maximizes $S(t_a, t_b, p)$ is to test every common ancestor and then select the one with the highest score. However, considering the total number of terms in GO, this approach is computationally expensive, especially for the computation of the genome-wide gene-to-gene similarities. Here, we propose and prove that only the *minimum common ancestors,* or MCA in short, need to be scanned to locate the best ancestor term. By dramatically reducing the number of parent terms to test, *NETSIM* can be efficiently computed.

Definition 1. Minimum Common Ancestor (MCA). *Let t_a and t_b be two Gene Ontology terms and P_{all} be the set of their common ancestors, a minimum common ancestor p is a term in P_{all} such that none of the descendants of p is in P_{all}.*

Two Gene Ontolgogy (GO) terms may have multiple common ancestors in GO and one ancestor may be the ancestor of another. For example in Figure 1A, t_i, t_j and *root* are the common ancestors of t_a and t_b, but only t_i and t_j are the MCAs. To compute term-to-term similarity with *NETSIM*, we prove that only the MCAs need be tested.

Lemma 1. *Let $S(t_a, t_b, p)$ be the term similarity between t_a and t_b via ancestor p with NETSIM, and p_1 and p_2 are two common ancestors of t_a and t_b. If p_1 is the MCA and is a descendant of $p2$, then $S(t_a, t_b, p_1) \geq S(t_a, t_b, p_2)$.*

Proof: Since p_1 is a descendant of p_2, we know that $U(t_a, t_b, p_1)$ is a subset of $U(t_a, t_b, p_2)$, G_{p1} is a subset of G_{p2}, but the values of G, $D(t_a, t_b)$, $h(t_a, t_b)$, G_a, and G_b are the same for the computation of both $S(t_a, t_b, p_1)$ and $S(t_a, t_b, p_2)$.

Given Equation 4, we have $0 \leqslant f(t_a, t_b, p_1) \leqslant 1$, and consquently $\frac{2\log|G|-2\log f(t_a,t_b,p_1)}{2\log|G|-(\log|G_a|+\log|G_b|)} \geq \frac{2\log|G|-2\log f(t_a,t_b,p_2)}{2\log|G|-(\log|G_a|+\log|G_b|)}$. And since G_{p1} is a subset of G_{p2}, we have $(1-\frac{h(t_a,t_b)}{|G|}\cdot\frac{|G_{p_1}|}{|G|}) \geq (1-\frac{h(t_a,t_b)}{|G|}\cdot\frac{|G_{p_2}|}{|G|})$.

Since both the first part and the second part of Equantion 3 via p_1 are greater than or equal to that via p_2, we have $S(t_a, t_b, p_1) \geqslant S(t_a, t_b, p_2)$. By Definition 1, for every non-MCA term p_2, there must exist at least one MCA p_1 satisfying $S(t_a, t_b, p_1) \geqslant S(t_a, t_b, p_2)$. Done.

Algorithm 1. *NETSIM*

Input: t_a, t_b: two terms in GO in the same category
NET: gene co-function network
GO: GO structure and annotations
Output: $Sim(t_a, t_b)$: similarity score between t_a and t_b

1: $P \leftarrow$ all MCAs of t_a, t_b in GO
2: $G_a \leftarrow$ all genes annotated to t_a and its descendants
3: $G_b \leftarrow$ all genes annotated to t_b and its descendants
4: $G \leftarrow$ all genes annotated to root and its descendants
5: $d \leftarrow D(G_a, G_b, NET)$
6: $SIM \leftarrow$ Empty
7: **for** every $p \in P$ **do**
8: $U \leftarrow$ path annotations from t_a or t_b to p
9: $f \leftarrow f(G_a, G_b, U)$
10: $h \leftarrow h(G_a, G_b, p)$
11: $S \leftarrow S(t_a, t_b, f, h, p)$
12: $SIM \leftarrow \{S\} \cup SIM$
13: **end for**
14: $Sim(t_a, t_b) \leftarrow$ maximum value in SIM

The pseudocode of *NETSIM* is shown in Algorithm 1. To locate the best MCA p that maximizes $S(t_a, t_b, p)$, we employ an iterative process to compute the term similarity via MCAs. It starts by locating all of the MCAs of t_a and t_b in GO, and saving them in P (line 1). Then, all the genes annotated to t_a (or t_b) and its descendants are saved in G_a (or G_b) (lines 2 and 3), and all of the genes annotated to the root are saved in G (line 4). Third, the dissimilarity betweens t_a and t_b is calculated based on Equation 1, and is saved in d (line 5). For each common ancestor p, we compute Path Annotation (line 8) and f (or h) based on Equation 4 (or Equation 5) (line 9 or 10). Next, we compute S based on Equation 3 (line 11), and append it to SIM (line 12). Finally, we choose the maximum value in SIM as the similarity score between t_a and t_b (line 14).

For the example in Figures 1A and 1C, both t_i and t_j are the MCAs of t_a and t_b. For t_i, we have $f(t_a, t_b, t_i)$=3.549, $h(t_a, t_b)$=3.784, $|U(t_a, t_b, t_i)|=|G_i|$=10, $|G|$=13 and D=0.280, then S(t_a, t_b, t_i)=0.687. For t_j, we have $f(t_a, t_b, t_j)$=3.392, $h(t_a, t_b)$=3.784, $|U(t_a, t_b, t_j)|$=8, $|G_j|$=11, $|G|$=13 and D=0.280, then S(t_a, t_b, t_j)=0.691. Finally, the similarity between t_a and t_b is $Sim(t_a, t_b)=S(t_a, t_b, t_j)$=0.691.
