# Supplementary figures and images for: Measuring semantic similarities by combining gene ontology annotations and gene co-function networks
Source: BMC Bioinformatics. 2015 Feb 14;16:44. doi: 10.1186/s12859-015-0474-7 (PMC4339680; doi:10.1186/s12859-015-0474-7)

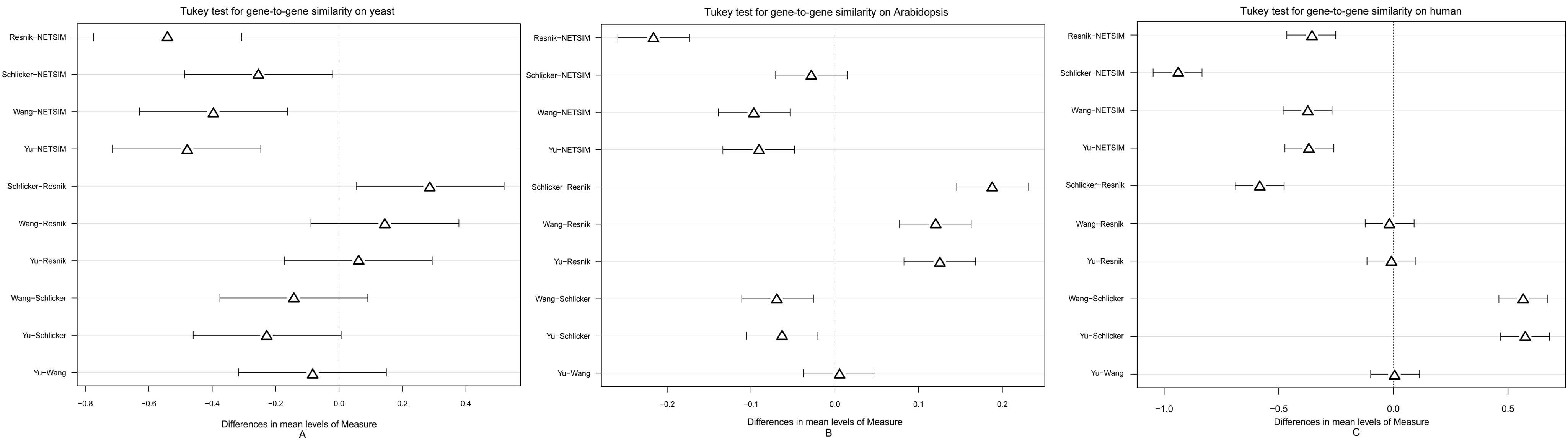

Supplement: Additional file 8: — Tukey test for gene-to-gene similarity on yeast (A), Arabidopsis (B) and human (C). For each line representing the difference of compared measures, the middle point (triangle) represents the difference in the observed means, and the line itself represents the range of the differences of compared measures. [file 12859_2015_474_MOESM8_ESM.tiff]

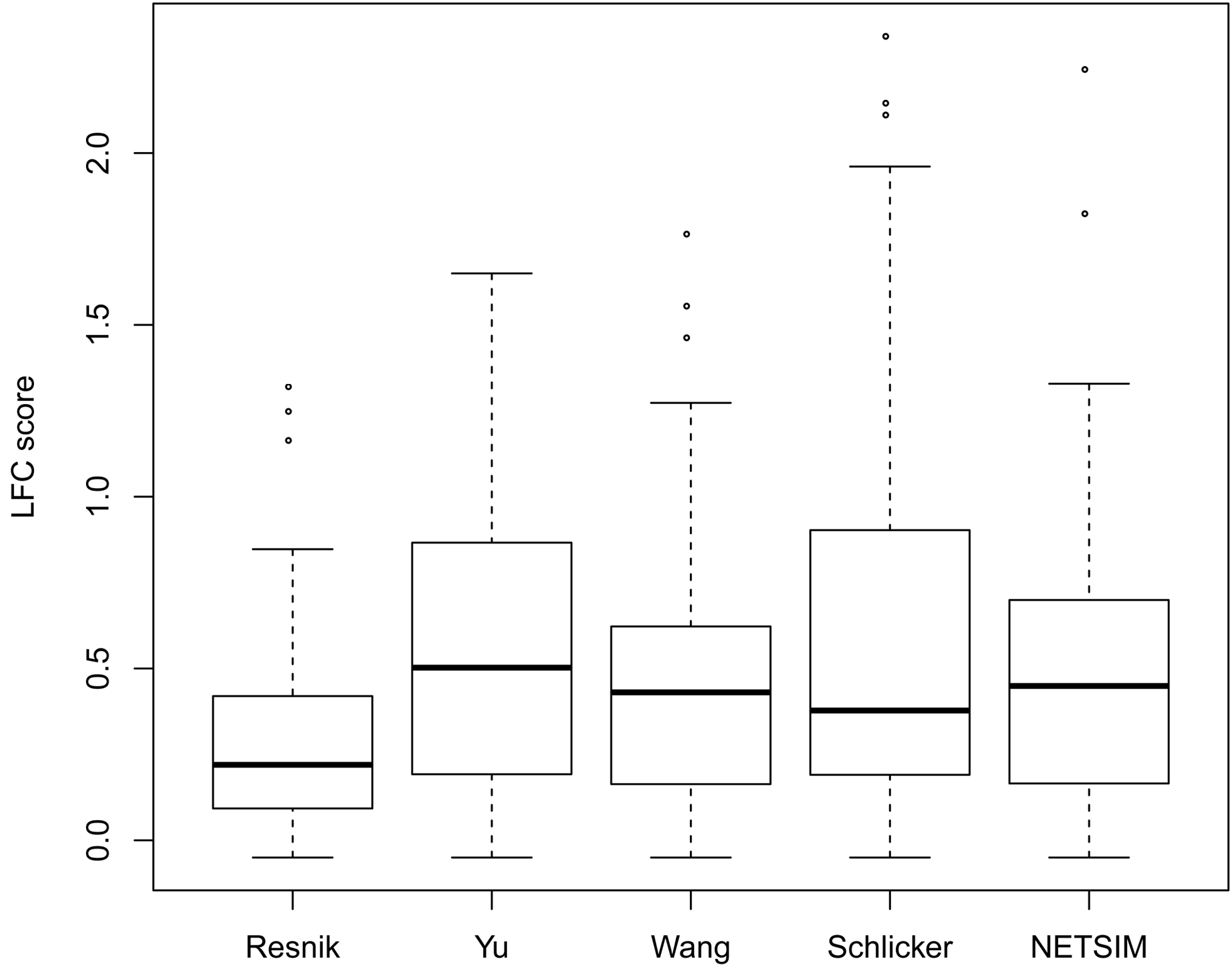

Supplement: Additional file 10: — Distributions of Log-transformed Fold Change (LFC) scores of similarity measures. Distributions of Log-transformed Fold Change (LFC) scores of similarity measures on GO’s biological process (BP) terms in yeast considering all annotations. [file 12859_2015_474_MOESM10_ESM.tiff]

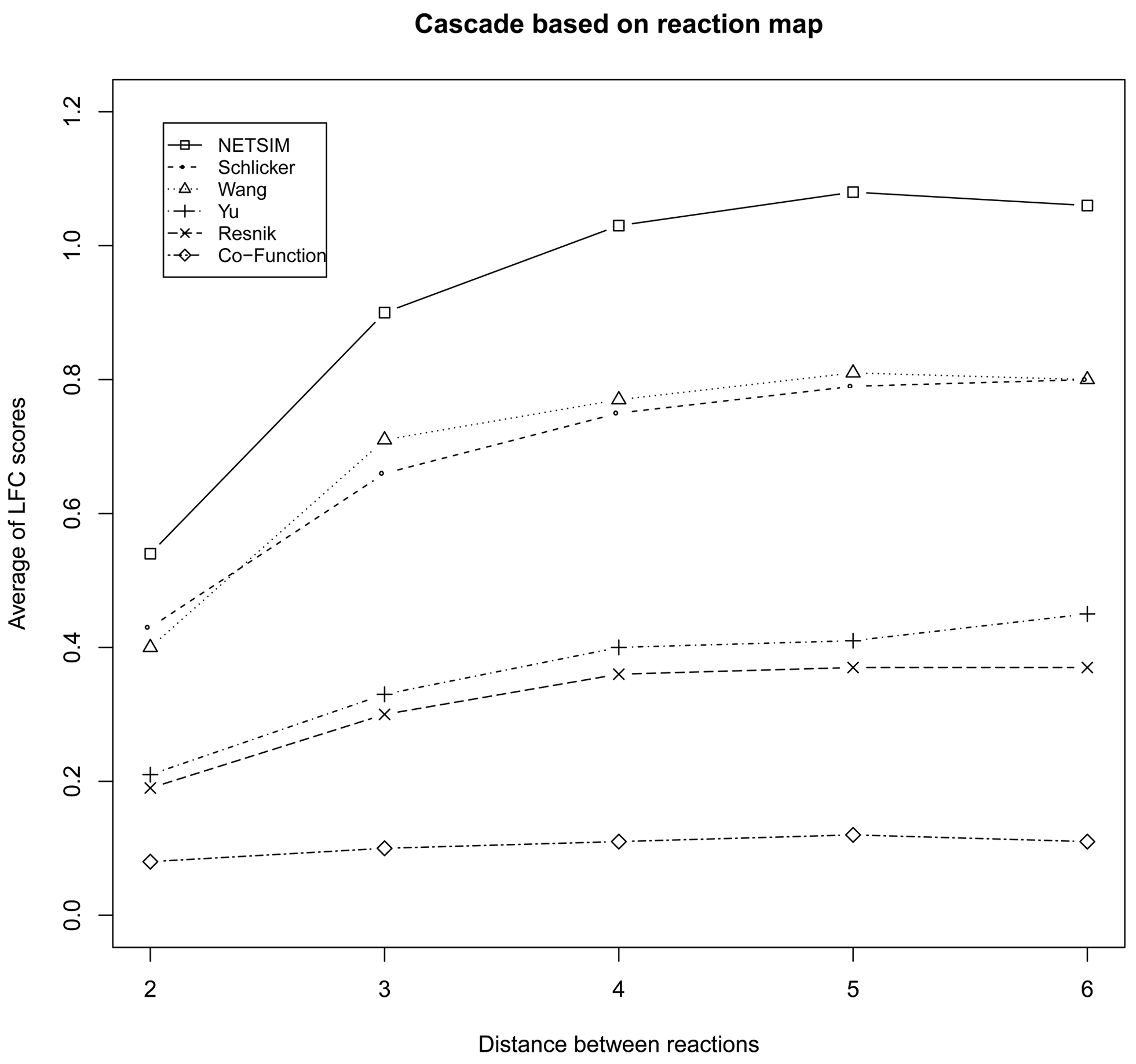

Supplement: Additional file 11: — The averaged Log-transformed Fold Change (LFC) scores of all the measures at different reaction path lengths on yeast. The x-axis is the path length to the given reaction. The y-axis is the average of LFC scores for all involved reactions. [file 12859_2015_474_MOESM11_ESM.tiff]

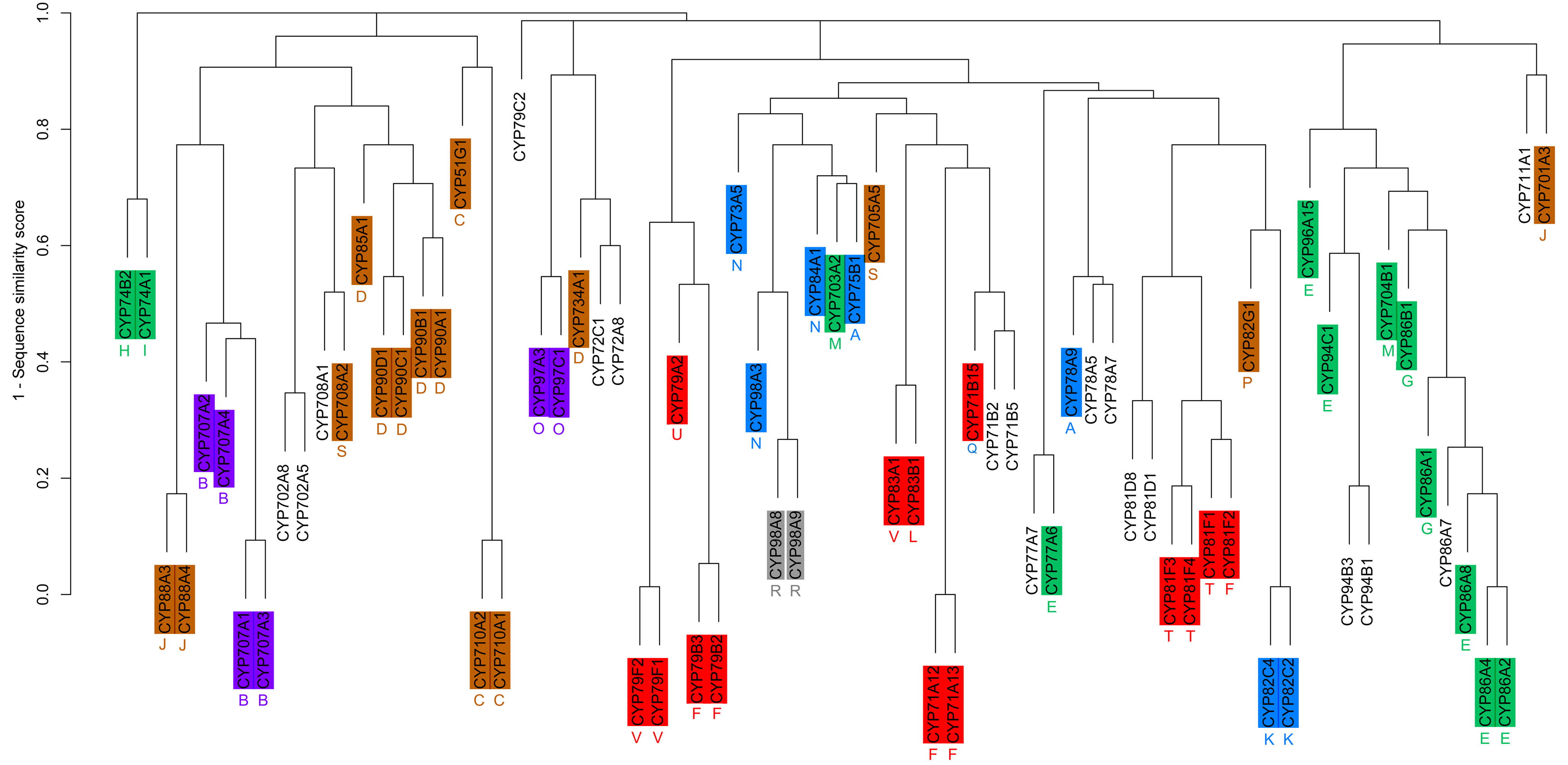

Supplement: Additional file 14: — Sequence based hierarchical clustering for Arabidopsis P450 gene family. The color coding is the same as Figure 6. The y-axis is 1 minus sequence-based gene-to-gene similarity score, which is the normalized percent identities value of function bl2seq in BLAST. [file 12859_2015_474_MOESM14_ESM.tiff]

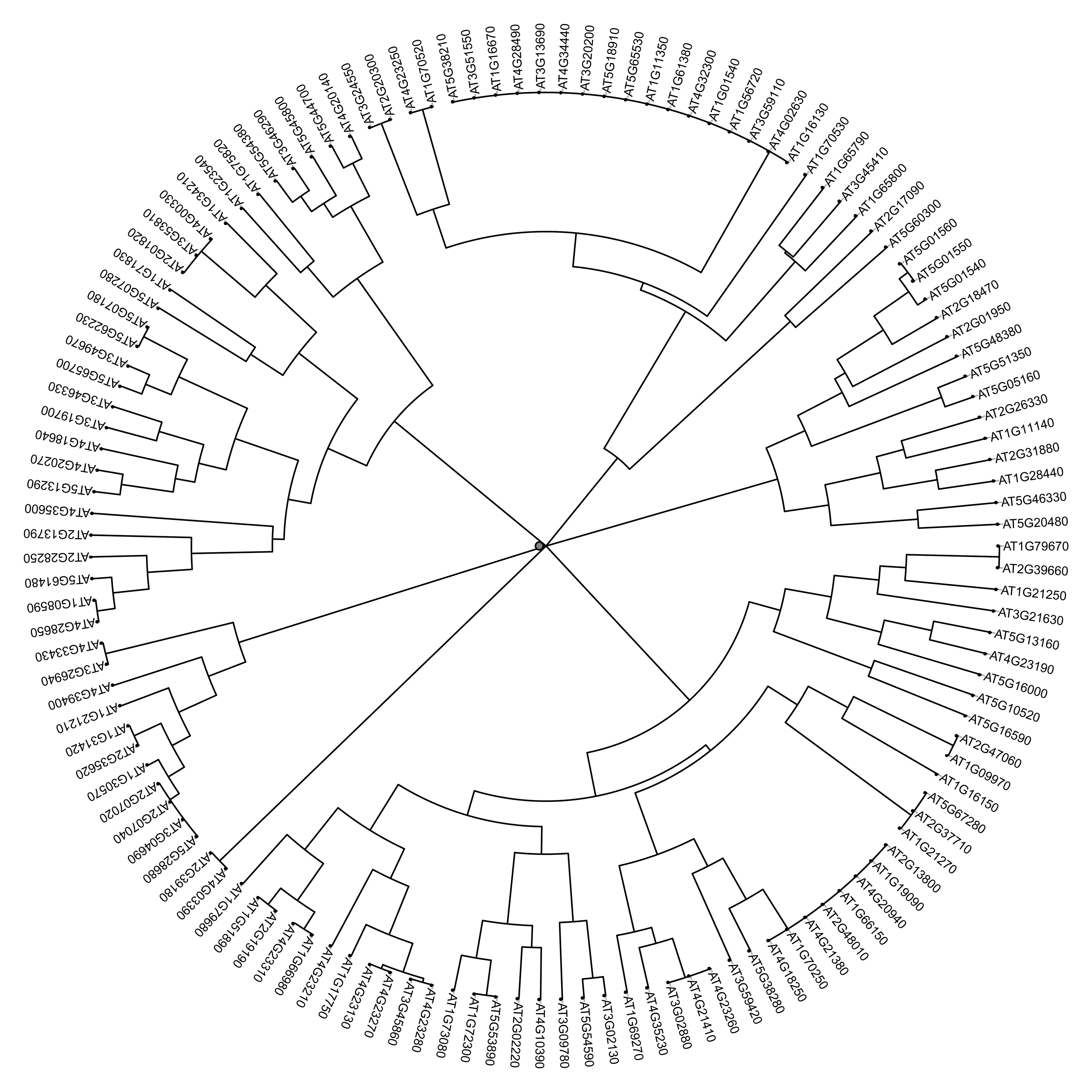

Supplement: Additional file 15: — NETSIM based clustering for Arabidopsis receptor-like kinase gene families (RLK) gene family. [file 12859_2015_474_MOESM15_ESM.tiff]

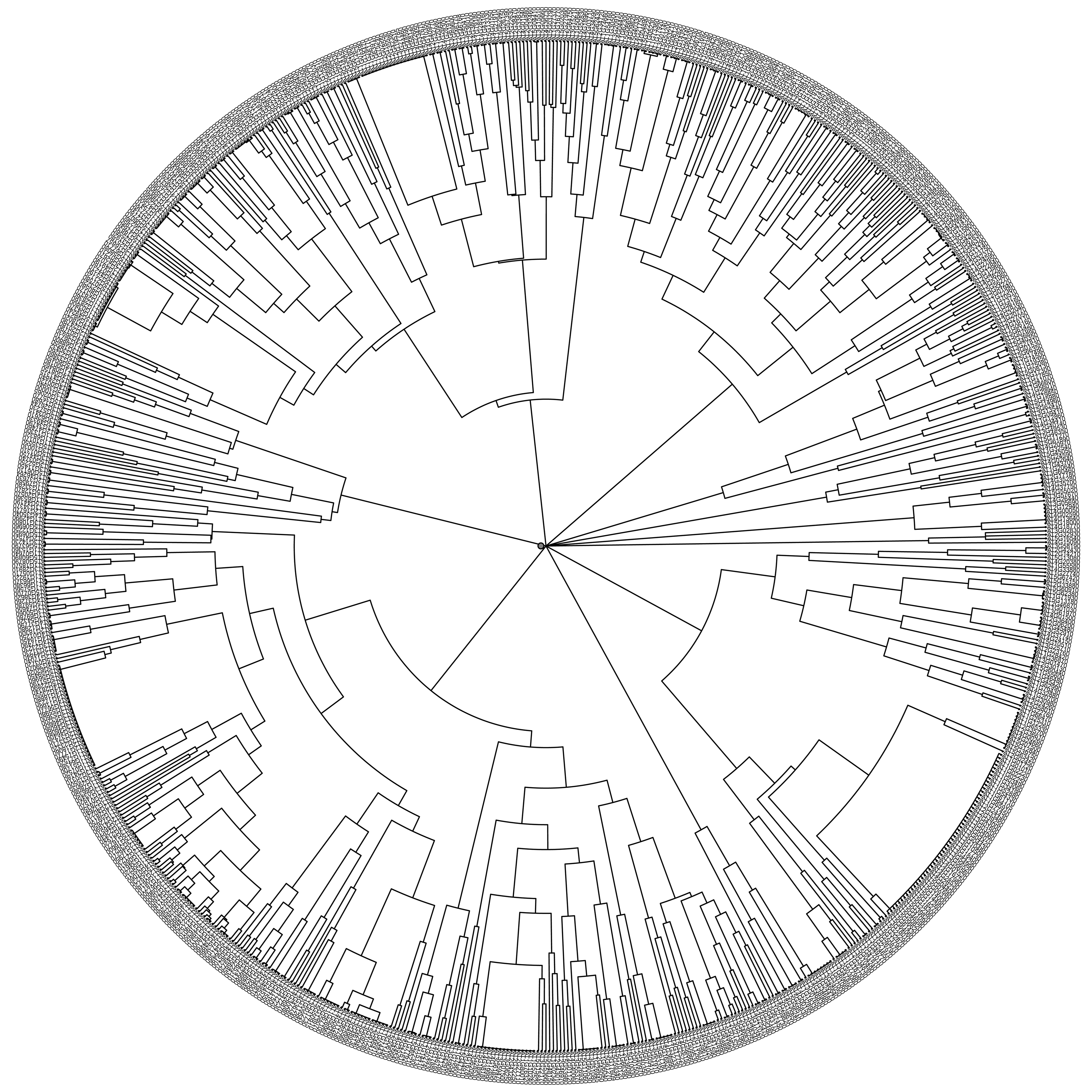

Supplement: Additional file 16: — NETSIM based clustering for Arabidopsis transcription factor families (TF). [file 12859_2015_474_MOESM16_ESM.tiff]

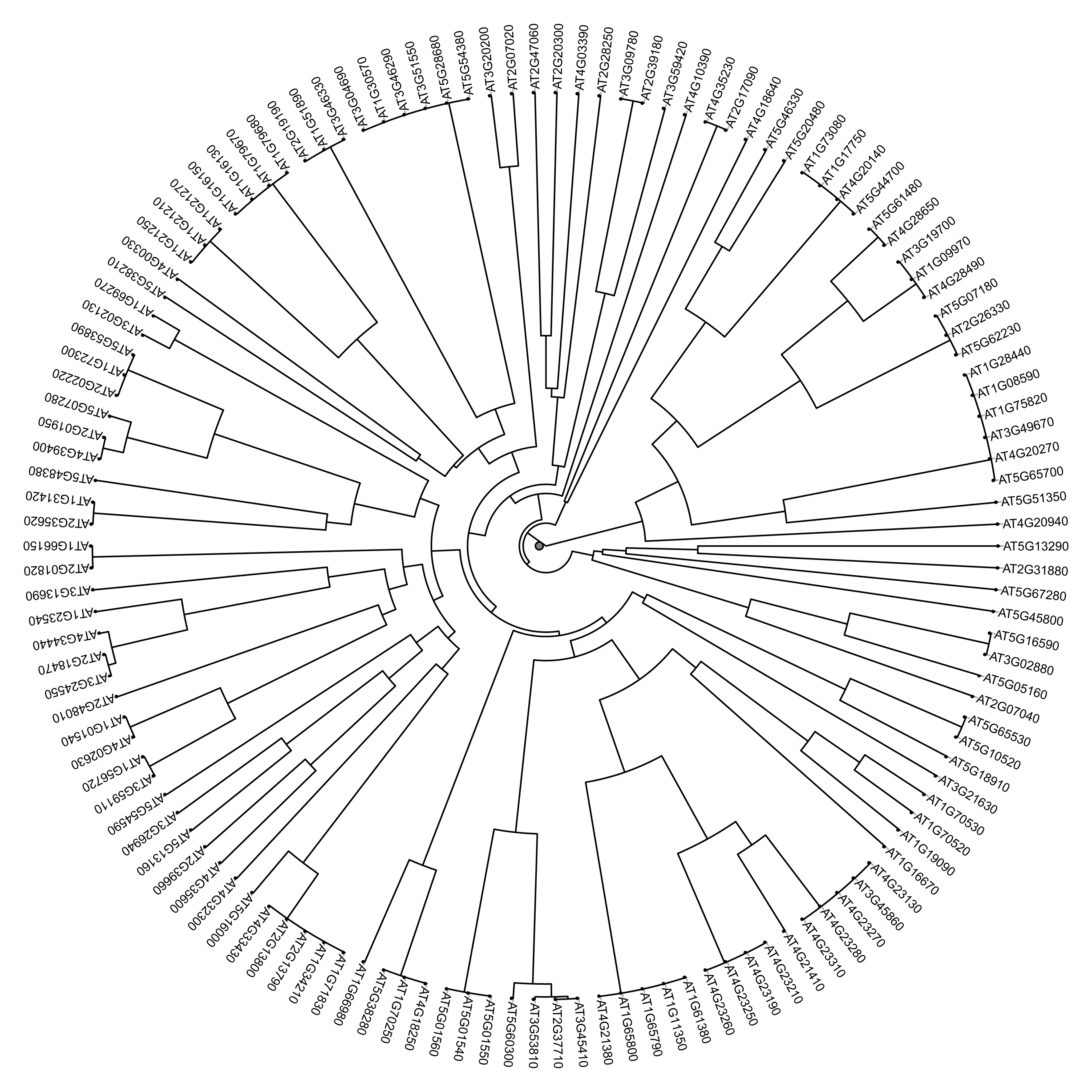

Supplement: Additional file 17: — Sequence based clustering for Arabidopsis receptor-like kinase gene families (RLK) gene family. The y-axis is 1 minus sequence-based gene-to-gene similarity score, which is the normalized percent identities value of function bl2seq in BLAST. [file 12859_2015_474_MOESM17_ESM.tiff]

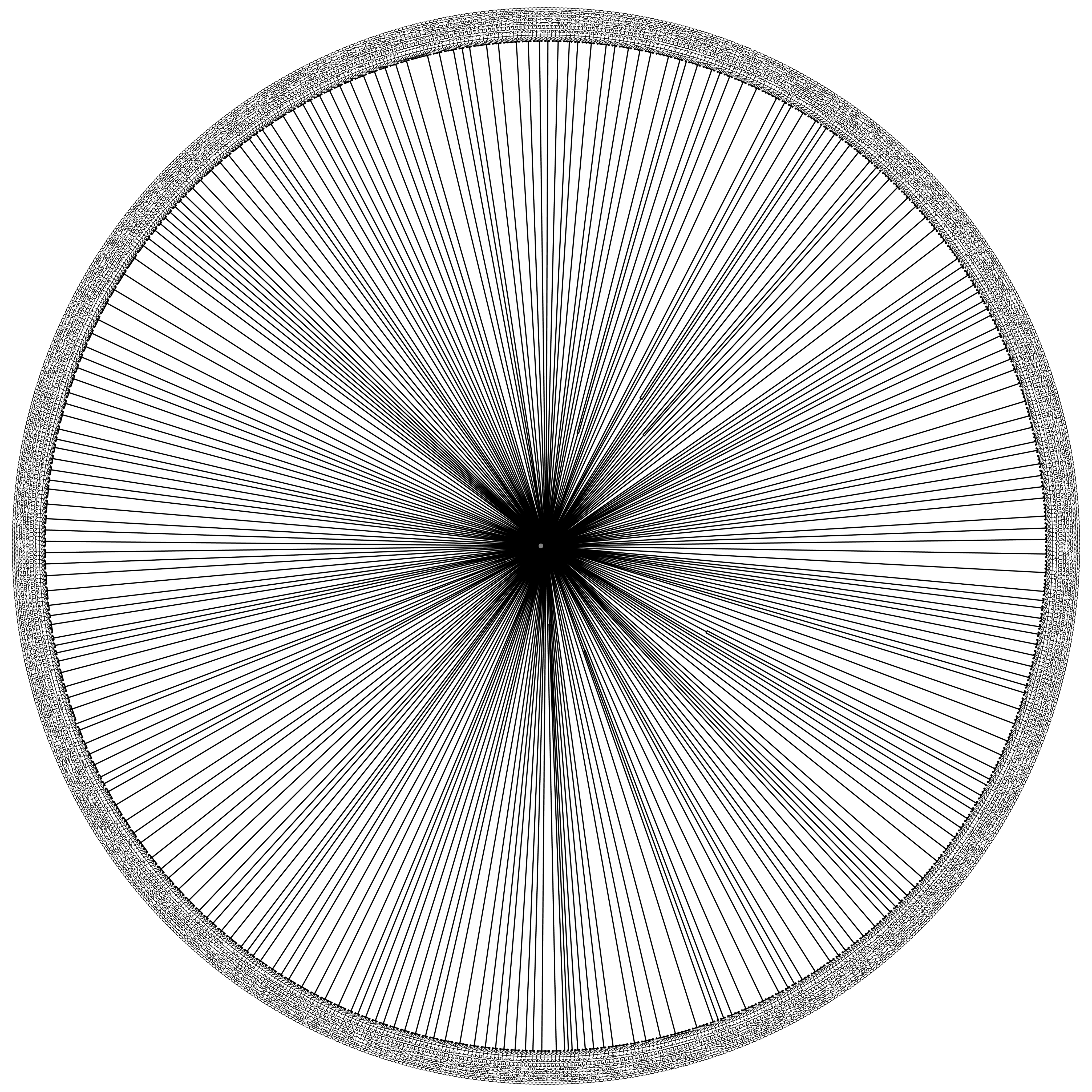

Supplement: Additional file 18: — Sequence based clustering for transcription factor families (TF) gene family. The y-axis is 1 minus sequence-based gene-to-gene similarity score, which is the normalized percent identities value of function bl2seq in BLAST. [file 12859_2015_474_MOESM18_ESM.tiff]
